# Transcatheter Closure of Patent Ductus Arteriosus in Premature Infants With Very Low Birth Weight

**DOI:** 10.3389/fped.2020.615919

**Published:** 2021-01-14

**Authors:** Jieh-Neng Wang, Yung-Chieh Lin, Min-Ling Hsieh, Yu-Jen Wei, Ying-Tzu Ju, Jing-Ming Wu

**Affiliations:** Department of Pediatrics, National Cheng Kung University Hospital, College of Medicine, National Cheng Kung University, Tainan, Taiwan

**Keywords:** preterm infant, low birth weight, patent ductus arteriosus, transcatheter, device closure

## Abstract

**Background:** The aim of this study was to describe our experience with transcatheter device closure of patent ductus arteriosus (PDA) in symptomatic low-birth-weight premature infants.

**Methods:** We performed a retrospective study of infants born with a birth body weight of < 2,000 g and admitted to National Cheng Kung University Hospital from September 2014 to December 2019. Basic demographic and clinical information as well as echocardiographic and angiographic data were recorded.

**Results:** Twenty-five premature infants (11 boys and 14 girls) born at gestational ages ranging between 22 and 35 weeks (mean, 25 weeks) were identified. The mean age at procedure was 34.5 ± 5.5 days, and the mean weight was 1,209 ± 94 g (range, 478–1,980 g). The mean diameter of the PDA was 3.4 ± 0.2 mm (range, 2.0–5.4 mm). The following devices were used in this study: Amplatzer Ductal Occluder II additional size (*n* = 20), Amplatzer Vascular Plug I (*n* = 1), and Amplatzer Vascular Plug II (*n* = 4). Complete closure was achieved in all patients. The mean follow-up period was 30.1 ± 17.3 months (range, 6–68 months). In total, 3 patients had left pulmonary artery (LPA) stenosis and 1 patient had coarctation of the aorta during the follow-up period. Younger procedure age and smaller procedure body weight were significantly associated with these obstructions.

**Conclusions:** Performing transcatheter PDA closure in symptomatic premature infants weighing more than 478 g is feasible using currently available devices; moreover, the procedure serves as an alternative to surgery.

## Introduction

Patent ductus arteriosus (PDA) is the most common cardiovascular abnormality in premature infants. The National Institute of Child Health and Human Development Neonatal Research Network reported that the incidence of PDA in preterm infants ranges from 20 to 60%, with an inverse relationship to birth weight (1). A PDA in preterm infants might cause considerable clinical sequelae including chronic lung disease, intraventricular hemorrhage, necrotising enterocolitis (NEC), congestive heart failure, and failure to thrive (2, 3). However, a consensus has not been reached on managing extremely low-birth-weight infants with a PDA, and practitioners do not know which infant will benefit from treatment (4). Therefore, “to treat or not to treat, when to treat, and how to treat” remain the main questions (5).

Hemodynamic significant PDAs are currently treated with a combination of supportive approaches, pharmacotherapy, and surgical ligation (6). Supportive management based on fluid restriction, and respiratory care is beneficial for most preterm infants; however, some infants still require further intervention (7, 8). Pharmacotherapy fails in 20–30% of premature babies (9, 10) and has some complications, such as renal insufficiency and bleeding (11). Surgical ligation of the PDA is invasive and associated with multiple adverse events including pneumothorax, phrenic nerve palsy, vocal cord paralysis, chylothorax, and scoliosis (3).

Although percutaneous closure of the PDA is among the safest of interventional cardiac procedures and is considered the procedure of choice for PDA closure beyond infancy (≥6 kg) (12), concerns have arisen regarding vascular access, transportation to the catheterization room, contrast administration, and lack of a suitable device (6, 13, 14). However, the introduction of new devices has led to the more frequent application of percutaneous PDA closure in premature babies (15–22). Although initial results have revealed that transcatheter closure of PDA in low-weight infants is feasible and is a potential alternative to surgery, several questions remain regarding the long-term results, the suitability of the transcatheter PDA closure for small infants of various weights, and the selection of the device. The purpose of this study was to report our approach to transcatheter closure of PDA in premature babies and the long-term outcomes.

## Patients and Methods

### Patients

We retrospectively reviewed the medical records of all low-birth-weight preterm infants who underwent transcatheter PDA closure at our institute from September 2014 to December 2019. In the initial stage from September 2014, our team offered the transcatheter method as an option in premature neonates with PDA weighting more than 1,200 g. From October 2016, we offered this procedure in every indicated patient. In our institution, the indication for PDA closure was the same as that for surgical ligation. Patients with symptomatic PDA, as evidenced by tachypnoea, failure to thrive, or being dependent on assisted ventilation, were evaluated for suitability of intervention. Medical intervention with indomethacin or ibuprofen was attempted in all these patients unless contraindicated. Echocardiography studies were performed with an iE-33 scanner (Philips Ultrasound, Bothell, WA, USA) and an S12 to 8 MHz sector transducer. PDA diameter, length, and morphology were defined by 2-dimensional and color Doppler echocardiography measurements. If PDA remained hemodynamically significant despite medical treatment, transcatheter closure or surgical ligation was offered to the parents who were fully informed regarding both procedures, their potential complications, alternative treatment options, and the team's past experiences. If in agreement, the parents provided written consent for the procedure chosen. This study was approved by our institutional review board.

### Procedural Technique

**Figure 1:**
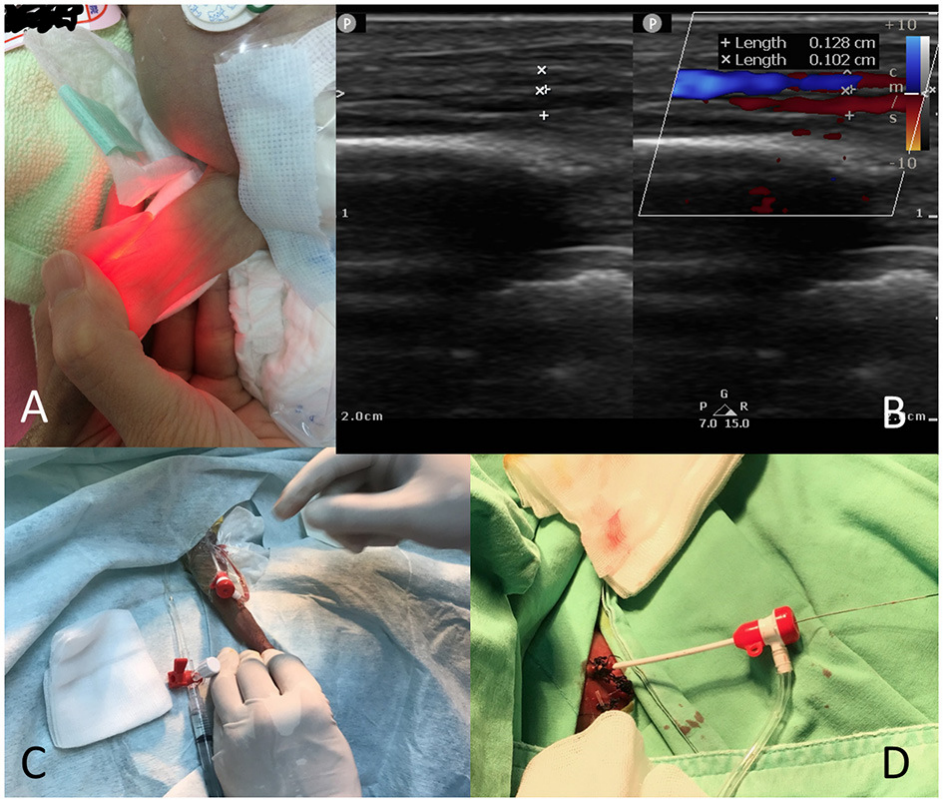
Preparation of venous cannulation. **(A)** Use of a transilluminator to identify the femoral vein location in a 533-g baby; **(B)** Use of ultrasound to identify the femoral vein in a 509-g baby; **(C)** A 4F sheath (femoral vein) in a 533-g baby; **(D)** A 4F sheath (umbilical vein) in a 478-g baby.

Because the patient had to be transferred from the neonatal intensive care unit (NICU) to the catheterization room, we opted not to perform elective intubation. We maintained the patient's ventilation mode in the NICU; however, if patients breathed room air or used a nasal cannula, we applied continuous positive airway pressure on them during the procedure because of the sedation. We used light sedation by intravenously administering midazolam (0.05–0.1 mg/kg) and antibiotic therapy with cephazolin (50 mg/kg). The neonates were transferred under an open warmer and were wrapped in a warm blanket (38°C) to maintain body temperature. Euthermia was maintained using a forced-air warming blanket and warm saline as the flush solution. A neonatologist, a nurse, and a respiratory therapist monitored the patient's condition during the procedure. For the preparation of venipuncture, a transilluminator (Red LED Light MK-02GX, ESU limited liability partnership, Kawasaki, Kanagawa, Japan) was initially used for landmark identification (Figure 1A). However, ultrasound assist with a linear ultrasound probe (L15-7io) with the Philips CX-50 system (Philips Medical Systems, Andover, MA, USA) were used from January 2019 (Figure 1B). Cannulation was performed via the femoral vein with a 22-gauge BD Insyte needle (Becton Dickinson Infusion Therapy Systems, Sandy, UT, USA) through which a 0.018-inch straight guidewire was introduced (Terumo, Tokyo, Japan) to allow insertion of a 4F Merit Prelude sheath (Merit Medical Systems Inc., South Jordan, UT, USA; Figure 1C). In patients with an umbilical vein catheter, we used a 0.018-inch straight guidewire to introduce the 4F sheath (Figure 1D).

Approach either from femoral vein or umbilical vein were the same. Our policy was “wire go first.” As far as possible, we avoided catheter manipulation within the cardiac chambers. A 4F Judkins right (JR) coronary catheter (Cordis, Fremont, CA, USA) was positioned at the inferior vena cava and right atrial junction. Under the lateral view monitoring, the catheter tip aimed to the front. A 0.025-inch Terumo guidewire (Terumo, Tokyo, Japan) was manipulated across the right ventricle, into the pulmonary artery, and across the PDA into the descending aorta (23). In patients weighing < 1,200 g, we steered a 0.014-inch Runthrough floppy guidewire (Terumo, Tokyo, Japan) across the right heart and placed it in the descending aorta to guide the JR catheter into the descending aorta (24). Hand injection ductal angiograms were performed in the lateral view and right anterior oblique views through the catheter positioned at the aorta end of PDA to define the PDA size. However, in very-low-birth-weight babies with potential renal function insufficiency, we did not perform contrast angiography. After the angiogram, we exchanged the JR catheter for an Amplatzer 4F Torque delivery sheath (St. Jude Medical, St Paul, MN, USA) or a 5F JR guiding catheter with a 0.035-inch J-curved wire (Boston Scientific, Marlborough, MA, USA).

### Device Selection

PDA morphology, size, and length were the key considerations in device selection. The Amplatzer Duct Occluder II with additional size (ADO-II-AS; Abbott, IL, USA) was suitable for patients with typical conditions. These devices with lengths of 2, 4, and 6 mm were used for 3–4, 4.1–6, and 6.1 mm or larger duct lengths, respectively. An occluder waist 1–1.5 mm larger than the measured PDA diameter was selected. However, for PDA diameter larger than 4 mm, the Amplatzer Vascular Plug I or II (AVP I or AVP II; Abbott, IL, USA) was used because of the waist-size limitation of the ADO-II-AS. If the ductus size exceeded 4 mm and the length was > 8 mm, the AVP II was used. Otherwise, the AVP I was used for lengths < 8 mm.

### Device Deployment

The device was prepared and advanced to the tip of the delivery sheath under fluoroscopic guidance. The sheath was returned to the aortic end of the PDA before deployment of the distal disc. We endeavored to deploy the aortic disc directly into the aortic ampulla of the ductus. The slow, controlled traction on the sheath resulted in deployment of the remaining central portion and pulmonary end of the device into the PDA. Again, we attempted to deploy as much of the device into the actual ductus and leave as little in the main pulmonary artery as possible. A detailed echocardiographic assessment including color Doppler flow in the PDA, left pulmonary artery (LPA), and descending aorta was performed prior to releasing the device from the delivery cable. When concern regarding obstruction of flow into the LPA or descending aorta arose, the device was repositioned using either gentle traction (in case of aortic obstruction) or partial recapture of the proximal device with redeployment in a slightly more distal location (in case of LPA obstruction). Upon confirmation that the device had completely closed the PDA, with no evidence of impingement on LPA or aortic flow, the device was released from the delivery cable and another echocardiographic assessment was conducted (15). After the procedure, we removed the sheath, restored haemostasis with manual compression, and transferred the babies back to the NICU.

### Results Assessment and Follow-Up

Immediate results were assessed using echocardiography in the cardiac catheterization laboratory. Color Doppler evaluation was performed to determine residual flows and identify any turbulence at the origin of the LPA and descending aorta. LPA and aortic obstruction were defined according to echocardiographic data as none (<1.5 m/s), mild flow acceleration (1.5–2 m/s), or stenosis/coarctation (>2 m/s) (25). Follow-up echocardiograms were performed at 1 week; at 1, 3, 6, and 12 months; and then yearly. Follow-up chest X-rays were performed the next day and at 1, 3, 6, and 12 months. To monitor the potential collateral damages, especially with regarding to intraventricular hemorrhage (IVH), brain echoes were performed before, 1 day, 1 and 2 weeks later after intervention.

### Statistical Analysis

Standard descriptive statistics (median with standard deviation for continuous variables) were calculated. Patients with each risk factor were compared with unaffected patients using Student's *t*-test, Wilcoxon rank, or chi-square tests, as appropriate. The threshold for statistical significance was set at *P* < 0.05. All statistical analyses were performed using SPSS version 17.0 (SPSS, Chicago, IL, USA).

## Results

From September 2014 to December 2019, 26 premature infants were initially enrolled for the transcatheter procedure. One patient, with a gestational age (GA) of 23 + 4 weeks and procedure body weight of 550 g, was excluded because of failure to set up the venous cannulation. In total, 25 patients were included in this study. The mean GA at birth was 25 weeks ± 6 days (range, 22 weeks ± 4 days to 35 weeks ± 1 day), and the mean birth weight was 1,351 ± 415 g (range, 421–1,772 g). The mean age at procedure was 34.5 ± 5.5 days (range, 2–118 days), and the mean body weight on procedure day was 1,209 ± 94 g (range, 478–1,980 g). All these premature infants had clinical complications related to different organ failures. They all had cardiomegaly, confirmed either through echocardiography or chest X-ray. Most patients were oxygen dependent (24/25), 12 patients required intubation with a mechanical ventilator (including 2 patients who required a high-frequency ventilator), and 8 patients received non-invasive ventilator support. Seven patients had developed pulmonary hemorrhage and 13 patients had bronchopulmonary dysplasia. In total, 23 of the neonates received at least 1 dose and up to 6 doses of ibuprofen therapy, and 5 patients had anuria after ibuprofen therapy. Two patients did not receive pharmacological therapy because of acute pulmonary hemorrhage. The basic demographic and clinical data are summarized in Table 1.

**Table 1:** Baseline patient demographic data.

**Patient Number**	**Birth weight (gram)**	**GA (week + day)**	**Sex**	**Procedure weight (gm)**	**Procedure age (day)**	**O2 demand**	**Ventilator setting**	Cadiomegaly^*^	**Ibuprofen course**	**Oliguria**	**Pulmonary hemorrhage**
1	1,174	28 + 5	M	1,950	48	1	NC	1	3	–	–
2	1,222	28 + 5	M	1,500	32	1	NC	1	3	–	–
3	1,330	31 + 1	F	1,400	23	1	NC	1	6	–	–
4	779	25 + 1	F	934	38	1	IMV	1	6	–	–
5	1,492	29 + 3	F	1,455	13	1	CPAP	1	–	–	POS
6	1,169	27 + 5	M	1,201	17	1	IMV	1	3	–	POS
7	766	26 + 3	F	836	36	1	IMV	1	6	–	POS
8	478	23 + 4	F	533	33	1	IMV	1	1	POS	–
9	1,705	30 + 6	F	1,505	17	1	NC	1	2	–	–
10	660	24 + 6	F	660	2	1	HFV, IMV	1	–	POS	POS
11	568	23 + 0	M	509	12	1	IMV	1	6	–	POS
12	462	22 + 4	M	478	7	1	IMV	1	1	POS	POS
13	1,772	35 + 1	F	1,980	22	–	–	1	3	–	–
14	1,442	29 + 2	F	1,558	9	1	HFV, IMV	1	0	POS	–
15	588	23 + 4	F	551	16	1	IMV	1	3	–	–
16	555	22 + 3	M	1,544	91	1	CPAP	1	3	–	–
17	1,330	34 + 1	M	1,343	7	1	CPAP	1	1	–	POS
18	490	22 + 5	F	1,245	64	1	NIPPV	1	6	POS	–
19	730	24 + 5	M	1,346	62	1	IMV	1	6	POS	–
20	653	23 + 6	M	1,374	57	1	NIPPV	1	6	–	–
21	979	26 + 4	F	1,518	40	1	NIPPV	1	6	–	–
22	592	23 + 4	M	657	20	1	IMV	1	6	–	–
23	598	23 + 4	F	796	30	1	IMV	1	6	–	–
24	421	23 + 2	F	1,947	118	1	CPAP	1	6	–	–
25	873	26 + 4	M	1,406	49	1	CPAP	1	6	–	–

* *cardiomegaly demonstrated either using echocardiography or chest X-ray*.

*CPAP, continuous positive airway pressure; F, female; HFV, high-frequency ventilation; IMV, intermittent mandatory ventilation; M, male; NC, nasal cannula; NIPPV, nasal intermittent positive pressure ventilation; POS, positive*.

**Figure 2:**
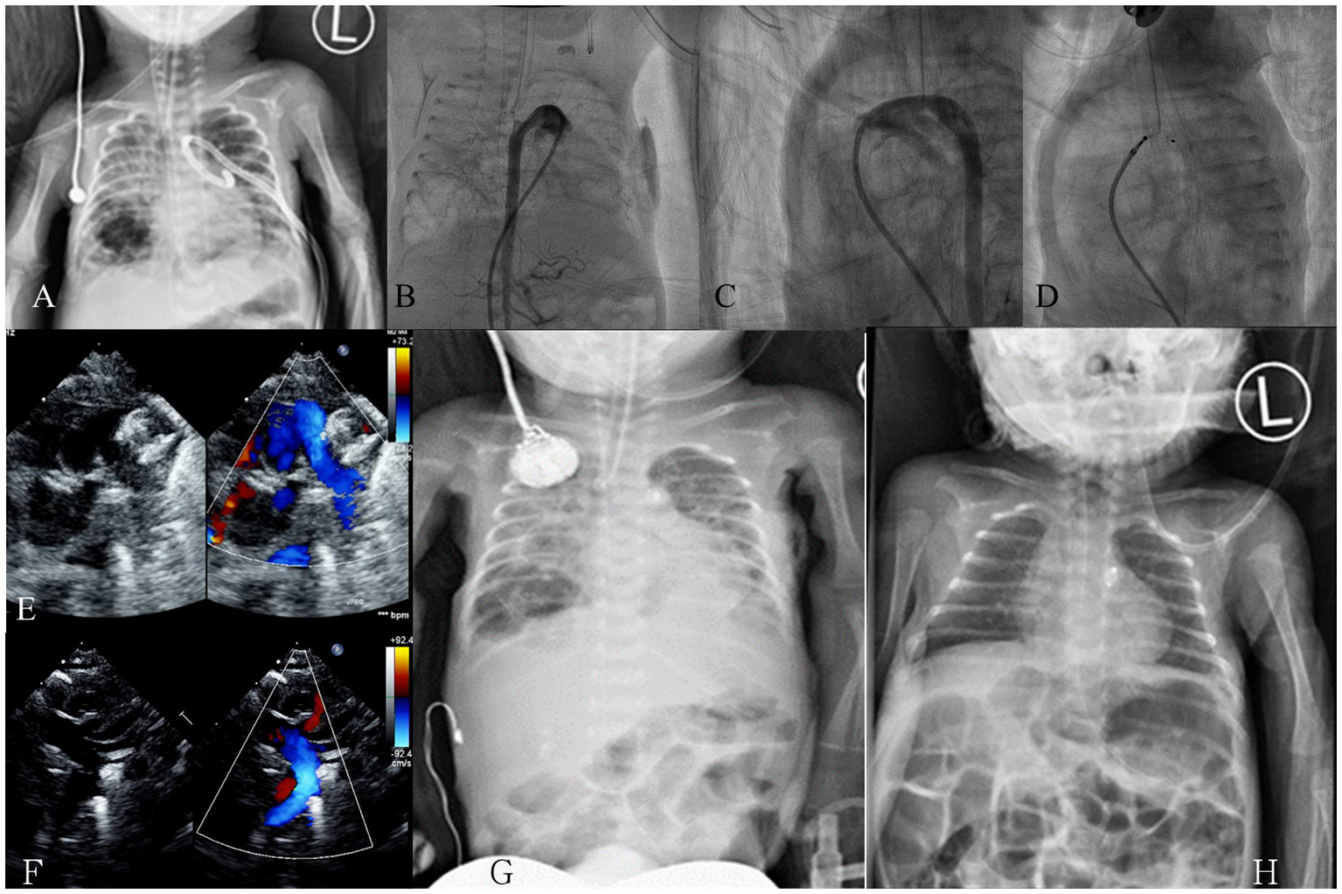
Case demonstration in a premature baby with a gestational age of 26 ± 3 weeks and a birth body weight of 766 g. **(A)** Chest X-ray on day 34 revealed poor lung condition with a left side pneumothorax post pigtail catheter; **(B)** On day 36 (body weight, 836 g), an anteroposterior view angiogram revealed a large tubular PDA; **(C)** Lateral view angiogram revealed a tubular type-C PDA, size: 2.9 mm, length: 6.4 mm; **(D)** An ADO-II-AS (4 × 4 mm) device was deployed on day 36; **(E)** Echocardiogram revealed device in position without pulmonary artery obstruction; **(F)** Echocardiogram revealed no evidence of coarctation of the aorta; **(G)** Follow-up chest X-ray on day 38 (2 days after the procedure) revealed rapid improvement; **(H)** Follow-up chest X-ray on day 70 (34 days after the procedure) revealed favorable condition. ADO-II-AS, Amplatzer Duct Occluder II with additional size; PDA, patent ductus arteriosus.

The traditional femoral vein approach and umbilical vein approach were used in 19 and 6 patients, respectively. The mean duration of the procedure was 42.8 ± 2.7 min (range, 22–83 min), and the mean fluoroscopy exposure duration was 12.9 ± 1.7 min (range, 4.1–39.2 min). The mean radiation exposure was 1278.9 ± 199.4 mGy/cm2 (range, 169–4,652 mGy/cm2). The mean diameter of the PDA was 3.4 ± 0.2 mm (range, 2.0–5.4 mm), and the mean length was 8.1 ± 0.6 mm (range, 4.0–16.5 mm). The most common PDA types were type F (10), type C (5), and type E (5) according to Phillip et al. (26) and Krichenko et al. (27) classification. After initial deployment and assessment, 11 patients required further adjustment. A total of 4 patients required a larger device, 2 patients required a shorter device, and 1 patient required a smaller device. In total, 25 occluders were successfully implanted. No device embolization was noted. The following devices were used in this study: ADO II-AS (*n* = 20), AVP I (*n* = 1), and AVP II (*n* = 4). Immediately following the procedure, most patients' oxygen demand rapidly decreased; therefore, assisted ventilation was withdrawn in the following few days, even in babies with pulmonary hemorrhage or poor lung condition (Figure 2). No complication was observed at the site of the venous puncture, including thrombosis or bleeding, and no sequelae were noted in the lower leg venous network at follow-up. The mean follow-up period was 30.1 ± 17.3 months (range, 6–68 months). Only 4 patients had minimal residual shunt at 1 week follow-up but all exhibited complete closure at 1 month follow-up. In total, 4 patients died during the follow-up period of this study. Mortality occurred between 2 and 163 days after the procedures. Two patients (case 6 and 14) died of NEC at 36 and 52 days, respectively. One patient (case 12) died 2 days after the procedure because of a severe fungal infection (Candida albicans) diagnosed before the intervention. We did not consider this infection catheter related because skin lesions were noted before the intervention, followed by eruption. Only one death (case 10), from progressive coarctation of the aorta (CoA), was related to the procedure; although the surgeon removed the device successfully at 150 days after the procedure, this patient died because of sepsis at 163 days after intervention. One patient (case 4) had mild to moderate tricuspid regurgitation which might have been related to the catheter procedure. In this case series, 2 patients had grade 3–4 intraventricular hemorrhage (IVH) already 2 weeks before closure, and one patient had grade 1 IVH before intervention. No progression were found in these 3 patients. Another 3 patients had grade 1–2 IVH found 20–50 days after closure. Procedure data and outcome summary are listed in Table 2.

**Table 2:** Catheterization data and outcome.

**Patient Number**	**PDA minimal diameter, mm**	**PDA length, mm**	**PDA type**	**Device**	**Outcome**	**Complications**	**Fluoroscopy time (minutes.seconds)**	**Radiation Exposure (mGY/cm2)**	**Approach**	**Follow up (months)**
1	3.0	11.2	Type F	ADO II AS (5 × 6)	Minimal -> closed		24.32	2,125	FV	68
2	3.6	10.7	Type F	Vascular Plug II (6 × 6)	Closed		19.47	1,785	FV	55
3	5.3	9.1	Type C	Vascular Plug I (8 × 7)	Minimal -> closed		30.51	2,988	FV	55
4	3.1	8.0	Type C	ADO II-AS (4 × 6)	Closed	TR	8.28	724	FV	43
5	2.9	8.5	Type F	ADO II-AS (4 × 6)	Closed		7.19	585	FV	42
6	2.4	7.6	Type C	ADO II-AS (4 × 6)	Closed		6.22	380	FV	36 days NEC Expired
7	2.6	5.4	Type C	ADO II AS (4 × 4)	Minimal -> closed		12.48	1,258	FV	40
8	3.5	5.7	Type E	ADO II AS (5 × 4)	Closed		18.24	1,863	FV	39
9	2.8	7.5	Type E	ADO II AS (5 × 4)	Closed		16.11	1,750	FV	38
10	2.9	5.3	Type C	ADO II AS (5 × 4)	Closed	CoA LPA	9.22	811	UV	163 days Sepsis post CoA operation expired
11	2.9	6.2	Type C	ADO II AS (5 × 4)	Closed	LPA	15.06	1,434	UV	38
12	2.0	4.0	Type C	ADO II AS (3 × 2)	Closed		10.22	715	UV	2 days Fungus sepsis expired
13	4.1	11.4	Type F	Vascular Plug II (6 × 6)	Closed		17.16	2,049	FV	32
14	3.4	10.1	Type C	ADO II-AS (5 × 6)	Closed		8.45	728	UV	52 days NEC Expired
15	2.2	4.5	Type C	ADO II AS (4 × 4)	Closed	LPA	10.14	843	UV	35
16	3.2	6.1	Type C	ADO II AS (5 × 4)	Closed		14.42	958	FV	27
17	5.0	12.4	Type F	Vascular Plug II (8 × 7)	Closed	LPA	39.22	4,652	UV	20
18	3.7	11.2	Type F	ADO II-AS (5 × 6)	Closed		4.47	397	FV	16
19	3.6	9.2	Type F	ADO II-AS (5 × 6)	Closed		11.31	1,480	FV	14
20	3.2	8.4	Type E	ADO II-AS (5 × 4)	Closed		14.49	1,980	FV	10
21	5.4	16.5	Type F	Vascular Plug II (8 × 7)	Closed		7.40	619	FV	8
22	3.1	5.2	Type E	ADO II-AS (5 × 2)	Minimal -> closed		4.21	771	FV	7
23	3.5	6.9	Type F	ADO II AS (5 × 4)	Closed		4.41	169	FV	7
24	3.5	7.2	Type F	ADO II AS (5 × 4)	Closed		5.37	652	FV	6
25	3.4	5.1	Type E	ADO II-AS (5 × 2)	Closed		4.09	256	FV	6

*ADO-II-AS, Amplatzer Duct Occluder II with additional size; AVP I, Amplatzer Vascular Plug I; AVP II, Amplatzer Vascular Plug II; CoA, coarctation of the aorta; FV, femoral vein; LPAS, left pulmonary artery stenosis; NEC, necrotizing enterocolitis; PDA, patent ductus arteriosus; TR, tricuspid regurgitation; UV, umbilical vein*.

In total, 3 patients had LPA stenosis and 1 patient had CoA; the overall incidence was 16% (4/25). We compared the risk factors for LPA and CoA, and the results are listed in Table 3. We found that younger procedure age and smaller procedure body weight were significantly related to these obstructions. However, the LPA stenosis was mild and < 20 mmHg during the follow-up period. One patient also had LPA hypoplasia initially but it gradually improved. Patients discharged from hospital were clinically stable without cardiovascular symptoms, except for one patient (case 10).

**Table 3:** Risk factors for left pulmonary artery obstruction or coarctation of the aorta.

**Risk factor**	**Yes (*n =* 4)**	**No (*n =* 21)**
Gender (male/female)	2/2	8/12
Gestational age (weeks)	26.7 ± 3.79	25.18 ± 2.85
Birth body weight (grams)	786.5 ± 364.4	937.2 ± 427.9
Age at procedure (days)	9.3 ± 6.1^*^	39.3 ± 27.6
Weight at procedure (grams)	765.8 ± 390.1^*^	1293.5 ± 441.9
PDA type	Type C (3), Type F (1)	type F (9), type C (2), type E (5)
PDA minimal diameter (mm)	3.3 ± 1.2	3.4 ± 0.8
PDA length (mm)	7.1 ± 3.6	8.3 ± 2.9
Device used	ADO II AS (3), AVP II (1)	ADO II AS (*n =* 17), AVP I (*n =* 1), AVP II (*n =* 3)

* *P < 0.05*.

*ADO-II-AS, Amplatzer Duct Occluder II with additional size; AVP I, Amplatzer Vascular Plug I; AVP II, Amplatzer Vascular Plug II; PDA, patent ductus arteriosus*.

## Discussion

Transcatheter closure of PDA is currently the standard procedure in most circumstances (28). However, performing this procedure in premature babies remains challenging because of body weight, vascular access, underlying physiological condition, device selection, and other potential problems. However, the most common problems encountered are similar to those for current therapy. Thus, surgical ligation of the PDA remains a safe and proven method for premature babies in the NICU (29). Bedside surgical ligation is considered the standard in current NICU care, which imposes a high standard of comparison. Related studies have not reported findings for patients weighing < 600 g. In this study, we revealed the feasibility of performing transcatheter PDA closure in symptomatic premature infants weighing more than 478 g, highlighting the procedure's potential as an alternative to surgery.

Sathanandam et al. (30) listed 10 crucial lessons from their experiences, from patient-related aspects to technique solutions. These difficulties emphasized the fact that the field of PDA closure in premature babies is still expanding. Device selection is the key problem in transcatheter closure. Device selection first depends on PDA anatomy (26, 27). The PDA seen exclusively in premature infants is often tortuous and distinctively long and wide relative to the diameter of the descending aorta (26). In this study, we also found that tubular type C and F are the most common types. According to these findings, the ADO-II-AS was the most common device employed in this study. This device is currently named the Amplatzer Piccolo Occluder (Abbott) and has been approved by the US Food and Drug Administration (31). However, this device is reserved for ductus with a diameter below 4 mm. The AVP I or II were reserved for bigger PDA. We did not routinely measure diameter by using angiography because of inaccurate measurements resulting from small amounts of hand injection and potential PDA spasms as well as to prevent contrast-induced nephropathy. The new Medtronic Micro Vascular Plug might be a promising device because it does not have any retention discs (20). However, the disadvantage of this device is less radio-opaque, rendering it more difficult to be visualized under fluoroscopy than using other devices (22).

Device-related LPA and aortic obstruction are consistent concerns after successful transcatheter closure of PDA in premature babies (15, 21, 25, 32). In the current study, 3 patients developed LPA stenosis and 1 patient had coarctation. In our study, LPA stenosis was associated with younger age and weight at procedure, which is similar to the findings of a related report (25). The most complicated case in this study (case 10) received PDA closure on day 2. Although the procedure was completed smoothly and the patient's initial condition was stable, the patient's condition progressively deteriorated. We believe this might have been due to unstable PDA status in premature infants in the first week.

Venous cannulation can be difficult in very small premature infants. Because the inner diameter of a 4F sheath is 1.33 mm, the minimum size of the femoral vessels in premature infants should be able to accommodate the sheath. However, according to our data, the femoral vein in a 509-g baby had a diameter of 1.3 mm (Figure 1B) and could tolerate the 4F femoral sheath during the procedure without complications. Al Hamod et al. (33) suggested the echo-guided puncture method in preterm babies; we adopted this method for our procedures from case 18 onward to facilitate the intervention. The umbilical vein approach is another alternative to neonatal cardiac catheterization (34). In this study, we demonstrated the use of the umbilical vein approach to close a PDA in a 478-g neonate. The artery approach should be avoided because of the potential complications (13).

Transportation to the catheterization lab for the procedure is a major concern and requires consideration of respiratory therapeutics and teamwork between neonatologists, nursing specialists, catheterization room staff, pediatric cardiologists, and interventionists. In this series, we used light sedation with nasal cannular or non-invasive ventilator support. However, some patients still required ventilator support because of poor lung condition (21). Two patients required a high-frequency ventilator and inhaled nitro oxide; however, we had to temporarily shift the high-frequency mode to mandatory ventilation mode during deployment for the procedure to be performed successfully.

Radiation exposure could be a concern in these small-sized babies. Therefore, we attempted to minimize radiation exposure and fluoroscopy time. We also avoided angiography to prevent contrast-induced nephropathy in 10 cases. We believe that as our experience grows, we can progressively minimize radiation exposure and time. Transcatheter closure of PDA in the NICU by using an echocardiography-guided cardiac catheter technique with minimal morbidity is becoming achievable and represents a considerable advance in neonatal care (35).

The limitations of this study are the relatively small sample size and the use of a single center. However, we have constructed a programme that applies currently available devices to perform transcatheter PDA closure in symptomatic premature infants weighing more than 478 g. This study also included longitudinal follow-up of up to 5 years or more. This is the first study using the umbilical vein approach to perform transcatheter PDA closure in premature babies. We therefore believe that body weight is not a major concern, and this procedure can be an alternative to surgery.

## Conclusions

Transcatheter PDA closure in symptomatic premature infants is feasible using currently available devices; moreover, the procedure serves as an alternative to surgery.

## Data Availability Statement

The original contributions presented in the study are included in the article/supplementary material, further inquiries can be directed to the corresponding author/s.

## Ethics Statement

The studies involving human participants were reviewed and approved by NCKUH (B-ER-108-334). Written informed consent to participate in this study was provided by the participants' legal guardian/next of kin.

## Author Contributions

J-NW contributed to the study design, collection of the study data, analysis and interpretation of the data, and drafting of the manuscript. Y-CL contributed to the study concept, analysis and interpretation of the data, and critical revision of the article. M-LH contributed to the collection of the study data, analysis and interpretation of the data, and critical revision of the article. Y-JW contributed to the collection of the study data and analysis of the data. Y-TJ contributed to the study design, statistical method, and analysis and interpretation of the data. J-MW contributed to the study design and concept and critical revision of the article. All authors contributed to the article and approved the submitted version.

## Conflict of Interest

The authors declare that the research was conducted in the absence of any commercial or financial relationships that could be construed as a potential conflict of interest.
